# Genetic diversity of *Echinococcus multilocularis* in red foxes from two Scandinavian countries: Denmark and Sweden

**DOI:** 10.1016/j.fawpar.2019.e00045

**Published:** 2019-02-27

**Authors:** Jenny Knapp, Gérald Umhang, Helene Wahlström, Mohammad Nafi Solaiman Al-Sabi, Erik O. Ågren, Heidi Larsen Enemark

**Affiliations:** a Department of Chrono-environnement, UMR UFC/CNRS 6249 aff. INRA, University of Bourgogne Franche-Comté, Besançon, France; b ANSES, Nancy Laboratory for Rabies and Wildlife, Wildlife Surveillance and Eco-epidemiology Unit, Technopôle Agricole et Vétérinaire, B.P. 40009, 54220 Malzéville, France; c National Veterinary Institute, SE-75189 Uppsala, Sweden; d National Veterinary Institute, Technical University of Denmark, Bülowsvej 27, 1870 Frederiksberg C, Denmark; e Department of Pharmacy, Faculty of Pharmacy, Al-Zaytoonah University of Jordan, Queen Alia Airport St. 594, P.O. Box 130, 11733 Amman, Jordan; f Norwegian Veterinary Institute, Department of Animal Health and Food Safety, P.O. Box 750 Sentrum, NO-0106 Oslo, Norway

**Keywords:** *Echinococcus multilocularis*, Scandinavian isolates, Genetic diversity, EmsB microsatellite

## Abstract

*Echinococcus multilocularis* is an endemic parasite of red foxes in several European countries. This parasite has been present for decades in central Europe i.e. Switzerland, Eastern France, Southern Germany and Austria, which constitute the core endemic area of Europe. In the Scandinavian countries Sweden and Denmark, several recent findings were made in foxes. To better understand the dynamics and geographic spread of *E. multilocularis* in Europe, genetic studies have been undertaken using the DNA microsatellite marker EmsB. In Europe, the parasite spread in hitherto non-endemic areas was suspected to take place after founder events, in which the core endemic area presents a wider genetic diversity in comparison to newly endemic areas. However, identical parasite profiles can be shared between them, highlighting the parasite spreading in a mainland-island system. In this study, Swedish (27 adult worms from seven red foxes) and Danish (38 adult worms from nine red foxes) isolates were examined using fragment size analyses of the tandemly repeated microsatellite EmsB in order to compare the genetic profiles of the Scandinavian worms with a reference collection of European worm isolates from seven countries. Six EmsB profiles were detected in the Scandinavian panel. Three profiles were described in Denmark and four in Sweden. Only one of these profiles was detected in both countries. All profiles identified in the present study have previously been found in other European countries, suggesting an epidemiological link. Due to the relatively low number of Scandinavian *E. multilocularis* isolates analysed so far, firm conclusions cannot be made regarding the true genetic diversity. Nevertheless, the low genetic variation detected in Sweden and Denmark in this study is similar to the values obtained from peripheral areas of the main European endemic focus, which were more recently colonized by *E. multilocularis*; and continuous surveillance of this parasite is warranted to provide further insight into its epidemiology in Scandinavia.

## Highlights

• EmsB diversity of 65 Danish and Swedish isolates of *E. multilocularis* is described.
• Six different EmsB profiles were found, all previously identified in Europe.
• This reflects a possible link between Scandinavia and other European countries.
• Only one of the six different EmsB profiles was shared between Sweden and Denmark.

## 1 Introduction

*Echinococcus multilocularis*, the small tapeworm of red foxes (*Vulpes vulpes*), is endemic in large parts of Europe (Oksanen et al., 2016). The prevalence of *E. multilocularis* in Europe has increased during the last decades and the geographical distribution has been reported to expand (Thompson et al., 2017; Karamon et al., 2014; Combes et al., 2012). In Southern Europe, *E. multilocularis* was reported in the Southern French Alps (Umhang et al., 2016) and in Northern Italy near the French (Massolo et al., 2018) and the Austrian borders (Manfredi et al., 2006). To the east, an expansion has been observed notably in Croatia (Beck et al., 2018), Hungary (Casulli et al., 2010) and Romania (Sikó et al., 2011). In the northwest, a geographical spread has been described in Belgium (Vervaeke et al., 2006), Netherlands (Takumi et al., 2008) and France (Combes et al., 2012). Lately, *E. multilocularis* was also found in the two Scandinavian countries; Denmark (Enemark et al., 2013; Saeed et al., 2006) and Sweden (Osterman Lind et al., 2011). In Denmark, *E. multilocularis* was first detected in 2000 in the Greater Copenhagen area (Saeed et al., 2006); yet, surveillance was not initiated until 2011 (Enemark et al., 2013). During a surveillance period of 5 years (2011–2015), infected foxes were found in three areas in Denmark: the Greater Copenhagen area (2000), Højer (2011 and ongoing) and Grindsted (2014) (Petersen et al., 2018; Wahlström et al., 2015). Surveillance of *E. multilocularis* was initiated in Sweden in 2000 following the first detection in Denmark, and was intensified after the first finding in Sweden in 2011 (Wahlström et al., 2012; Wahlström et al., 2011). At present, *E. multilocularis* has been found in the four Swedish counties: Västra Götaland, Södermanland, Dalarna and Kronoberg (Miller et al., 2016; Wahlström et al., 2011). The national prevalence in red fox in Denmark and Sweden is low, approximately 1% and 0.1%, respectively (Wahlström et al., 2015). However, in certain areas the prevalence has been reported to be higher, up to 1% in Sweden and up to 30% in Denmark (Petersen et al., 2018; Wahlström et al., 2015). In Sweden, it is unknown if the parasite has been introduced recently or if it has been present for a long time at a prevalence below the detection level of the monitoring. Likewise, it is unknown if high endemic foci have existed in Denmark for many years or if this is a relatively new event. In areas where the parasite has been introduced and established recently the prevalence is likely to increase in the next decades (Karamon et al., 2014) and thus, exposure to humans may increase over time.

Genotyping has the potential to link newly introduced cases to old cases from endemic areas, in particular if highly polymorphic targets such as microsatellites are used (Freeland et al., 2011). The EmsB microsatellite marker has been used to describe genetic profiles of *E. multilocularis* isolates from a range of geographic regions including Europe (Knapp et al., 2008, Knapp et al., 2009, Knapp et al., 2012; Umhang et al., 2014; Umhang et al., 2017). In order to assess and compare the genetic diversity of *E. multilocularis* isolates the EmsB marker appears as a relevant tool (Bart et al., 2006), with an available collection of genotyped samples gathered in a database (Knapp et al., 2017). Indeed, microsatellites have a higher mutation rate than mitochondrial or nuclear target (Freeland et al., 2011) and therefore the EmsB microsatellite has shown potential to uncover low genetic diversity e.g. in areas where the parasite was recently introduced or in a defined geographical area such as in *E. multilocularis* infected rodents collected from the same field (Knapp et al., 2007). By using the EmsB marker, larger genetic diversity was found in the European historical endemic core areas of *E. multilocularis* than in new endemic areas. In the canton of Zurich (Switzerland), Northern Austria, Jura Swab (Germany) and Eastern France, 8 to 19 different EmsB profiles were described, whereas in new endemic areas such as Northern Poland, Central Slovakia, Northern and Western France only 3 to 6 different EmsB profiles have been found (Umhang et al., 2014; Knapp et al., 2009). Moreover, most (60%) of the EmsB profiles detected in the newly endemic areas in Europe have previously been identified in the historical endemic core area (Knapp et al., 2009), suggesting that the newly endemic areas were supplied by parasites from the historical endemic area.

In this study, we use the EmsB marker to genotype available *E. multilocularis* worms from Denmark and Sweden collected during 2010–2014. The aims were to further characterize the *E. multilocularis* isolates from these countries and to compare the genetic profiles with findings from other European regions to clarify if similar subtypes have been identified in other regions and to describe the within-country genetic diversity in Denmark and Sweden and compare it to findings in other European countries. Overall, we aimed at generating data that may be used to increase knowledge of the transmission dynamics of *E. multilocularis* in Europe.

## 2 Materials and methods

### 2.1 Sampling of foxes and collection of *E. multilocularis* worms

The foxes from Denmark included in the present study were collected during the period September 2012 to January 2014 as part of the Danish surveillance program. The program, which was initiated in September 2011, had included 1040 red foxes (*Vulpes vulpes*) and a total of 1500 wild carnivores until November 2014. The majority of the foxes included in the surveillance were hunted from all over the country by the Danish Nature Agency or by voluntary hunters (Petersen et al., 2018; Enemark et al., 2013). Prior to analysis by the sedimentation and counting technique (Eckert, 2003) the foxes were frozen at -80 °C for at least 4 days. Identification of *E. multilocularis* worms was based on the morphological characteristics of scolex and proglottids (Eckert, 2003), and the identification was verified by PCR (Stefanić et al., 2004). All positive foxes from Denmark (*n* = 10) from which material for subtyping was available, were included in the present study.

After the first positive red fox was identified in Sweden in February 2011, hunted foxes were collected in two studies. During 2011, 2985 foxes were submitted for *E. multilocularis* screening (Wahlström et al., 2012) and from 2012 to 2014 up to 30 foxes were submitted from each of the four counties where *E. multilocularis* had been discovered. Foxes within a radius of 20 km from the site of the first positive sample were included in the study. All foxes were georeferenced. Prior to analysis, foxes were frozen at -80 °C for at least 5 days. Foxes were analysed by the segmental sedimentation and counting technique (SSCT) (Umhang et al., 2011). Morphologically identified worms were confirmed by PCR (Isaksson et al., 2014). All *E. multilocularis* positive foxes from Sweden (*n* = 7) identified until October 2013 were included in this study.

From each positive fox, five to seven worms were selected for genotyping. However, seven foxes had less than five worms and therefore all worms i.e. 1–4 were genotyped from these animals (Table 1).

**Table 1:** Descriptive data regarding the analysed *Echinococcus multilocularis* worms recovered from red foxes in Denmark and Sweden, 2010–2014.

Country	Country/province	Sampling year	Fox code	EmsB profile (*no of worms*)	Corresponding European profiles^a^
Denmark	H	2012	DK1H	P4(1)	G23/G27/G28
Denmark	H	2012	DK2H	P4(*4*)	
Denmark	H	2012	DK3H	P4(*2*)	
Denmark	H	2013	DK4H	P4(*3*)	
Denmark	H	2013	DK5H	P4(*5*)	
Denmark	H	2013	DK6H	P4(*5*)	
Denmark	H	2014	DK7H	P4(*6*)	
Denmark	H	2013	DK10H	b(*2*)	Unvalidated profile
Denmark	G	2013	DK8G	P1(*6*)	G06/G07
Denmark	G	2014	DK9G	P5(*6*)	G19
Sweden	VG	2010	SE1VG	P6(*5*)	G20/G21
Sweden	VG	2011	SE2VG	P6(*1*)	
Sweden	VG	2012	SE3VG	P6(*7*)	
Sweden	VG	2012	SE4VG	P6(2)	
Sweden	D	2011	SE5D	P2(*1*) P3(*2*) b(*4*)	G05/G07 G07 Unvalidated profile
Sweden	S	2011	SE6S	P1(*5*) P2(*2*)	G06/G07 G05/G07
Sweden	S	2013	SE7S	P1(*2*) b(*4*)	G06/G07 Unvalidated profile

H: Højer; G: Grindsted; VG: Västra Götaland, D: Dalarna, S: Södermanland.

a Knapp et al., 2009.

b Samples excluded from the present study due to lack of compliance with the technical validation requirements.

### 2.2 DNA extraction and PCR

The worms were stored in 70% (v/v) ethanol until molecular analysis. In all but five worms, individual DNA extraction was done using the High Pure PCR Template Preparation kit® (Roche Diagnostics, Mannheim, Germany) and purified DNA was eluted in 200 μl of the provided elution buffer. DNA extracts were stored at −20 °C prior to PCR analysis. DNA of five Swedish isolates from three foxes, SE5D (two of seven worms), SE6S (two of seven worms) and SE2VG (one worm) (Table 1), was extracted using a Magnatrix 8000+ robot® and NorDiag Vet Viral NA extraction kit®, according to the manufacturer's instructions (NorDiag AB, Hägersten, Sweden).

A fluorescent PCR assay was carried out on DNA from each worm (Knapp et al., 2007; Umhang et al., 2014). Briefly, the reaction was performed in a 25 μl reaction mixture, containing 200 μM of each dNTP, 0.4 μM of fluorescent forward primer EmsB A, 0.7 μM of reverse primer EmsB C, and 0.5 U of Platinum Taq DNA Polymerase enzyme® (Life Technologies, CA), with the addition of Platinum 1 and PCR Buffer® (Life Technologies, CA). The amplification reaction was performed in a Veriti thermal cycler® (Life Technologies, CA), by a pre-amplification step of 94 °C for 2 min, followed by 45 cycles with a denaturing step at 94 °C for 30 s, annealing at 60 °C for 30 s, and extension at 72 °C for 1 min, with a final elongation at 72 °C for 45 min.

### 2.3 Microsatellite analysis and assessment of genetic diversity

Fragment size analysis was performed by capillary electrophoresis of PCR products performed on an automaton sequencer machine (ABI Prism 310; Life Technologies, CA). The size (base pair) and height (fluorescence intensity) of each peak of the EmsB electrophoregrams were obtained with the use of GeneMapper 4.1® (Life Technologies, CA). To normalize fluorescence intensity, each fluorescence peak value was divided by the sum of all fluorescence values obtained for all peaks of a given sample (peaks retained are above 10% of the highest peak; see Knapp et al., 2007).

Genotyping by EmsB was based on a microsatellite with 40 copies on the same chromosome (Valot et al., 2015) and determination of profiles was performed by quantitative analysis of the height of the peaks from each allele in the electrophoregram (Knapp et al., 2007). The technical validation of each *E. multilocularis* electrophoregram profile was based on a multi-criteria approach including reproducibility (each sample performed in duplicate), global electrophoregram aspects (number and distribution of peaks), and genetic distance from other existing *E. multilocularis* EmsB profiles from the same geographical region. Samples that could not be technically validated based on these criteria were excluded from the genetic analyses.

In order to describe the genetic relationship among the worms, the samples were grouped into different EmsB profiles, defined by performing hierarchical clustering analysis using the Euclidean distance and the unweighted-pair group method with average linkages (UPGMA method) (Legendre and Legendre, 2012). The stability of the identified clusters was tested by a multiscale bootstrap resampling (Bt = 1000), resulting in approximately unbiased (au) *P*-values. A dendrogram was built based on results from the hierarchical clustering analysis using the package “pvclust” available under the R Project (v 3.0.0) (R Development Core Team, 2016). The worms were considered to belong to different profiles if the Euclidean distance between the samples was >0.08 (Knapp et al., 2007). Two *E. granulosus* sensu stricto (G1) were used as an outgroup in the present dendrogram.

In order to evaluate the genetic relationship with other previously genotyped *E. multilocularis* isolates, each EmsB profile identified was compared with a reference collection of profiles previously obtained by Knapp et al., 2007, Knapp et al., 2008, Knapp et al., 2009 and Umhang et al., 2017 and typed by EmsB from both European and non-European countries hereafter named the “ref collection” (Knapp et al., 2017). This collection included 49 EmsB profiles obtained from 1058 worm isolates from 269 red foxes, isolated in France (number of genotyped worms = 566; number of foxes = 162), Switzerland (84; 19), Germany (87; 18), Austria (98; 22), Slovakia (63; 14), Poland (94; 20) and Czech Republic (66, 14) (Knapp et al., 2009; Knapp et al., 2008; Knapp et al., 2007; Umhang et al., 2017; Umhang et al., 2014). The non-European collection included seven profiles from 54 worms originating from Asia (*n* = 13), North America (*n* = 14) and the archipelago of Svalbard, Norway (*n* = 27). The comparison was done by evaluating the genetic distance of one sample from each profile obtained in this study with all the samples from the collection using the R statistical software (R Development Core Team, 2016) as described in the EmsB Guidelines from the EWET database available online (https://ewet-db.univ-fcomte.fr) (Knapp et al., 2017). Samples with a genetic distance ≤0.08 are considered belonging to the same EmsB profile.

## 3 Results

A total of 74 worms from 17 foxes were genotyped. Of these, 65 worms from 16 foxes were successfully genotyped: 38 worms from nine Danish foxes and 27 worms from seven Swedish foxes (Table 1). Two worms from one fox from Denmark (DK10) and eight worms from two Swedish foxes (SE5D, SE7S) were excluded as they did not fulfil the technical validation criteria.

**Fig. 1:**
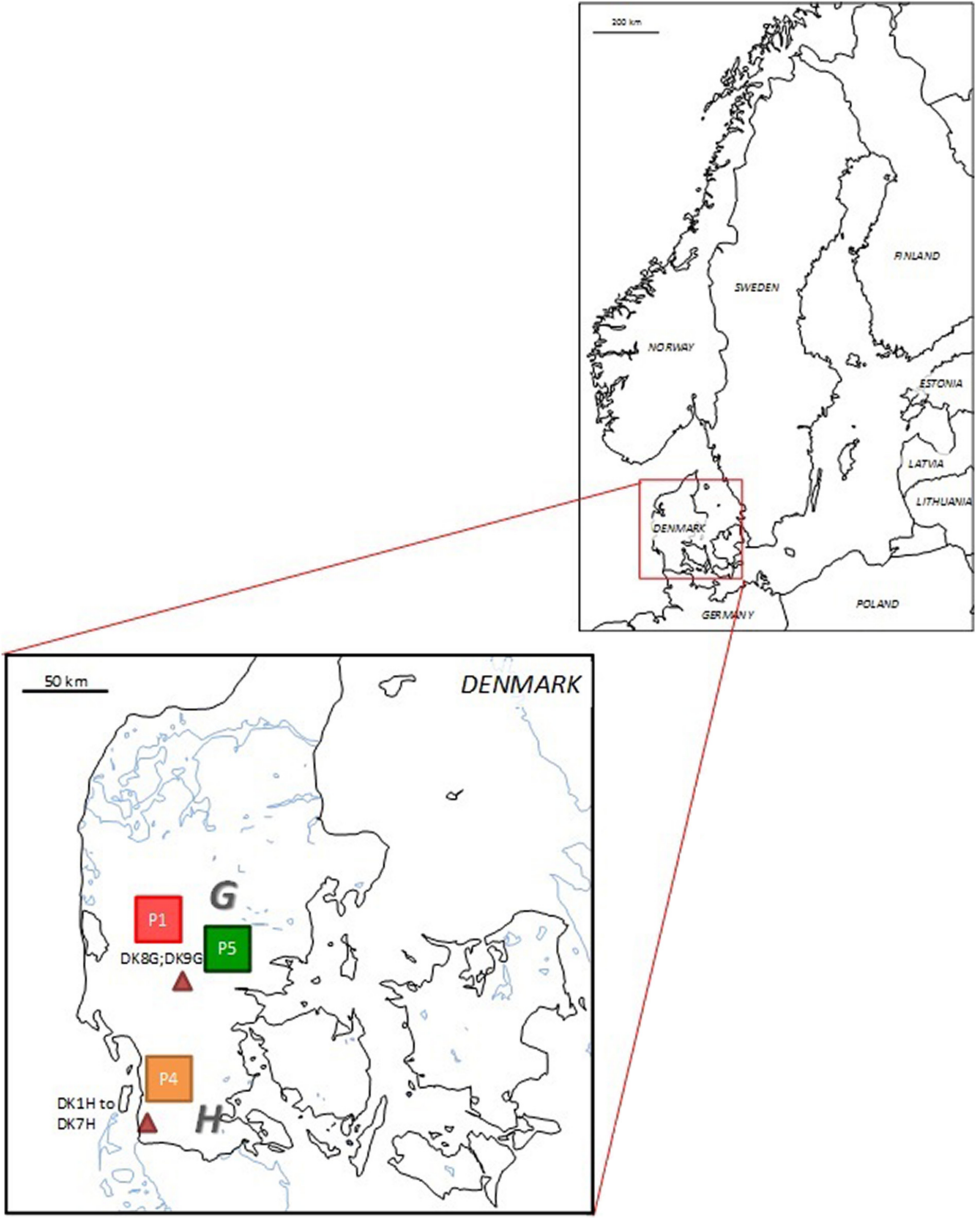

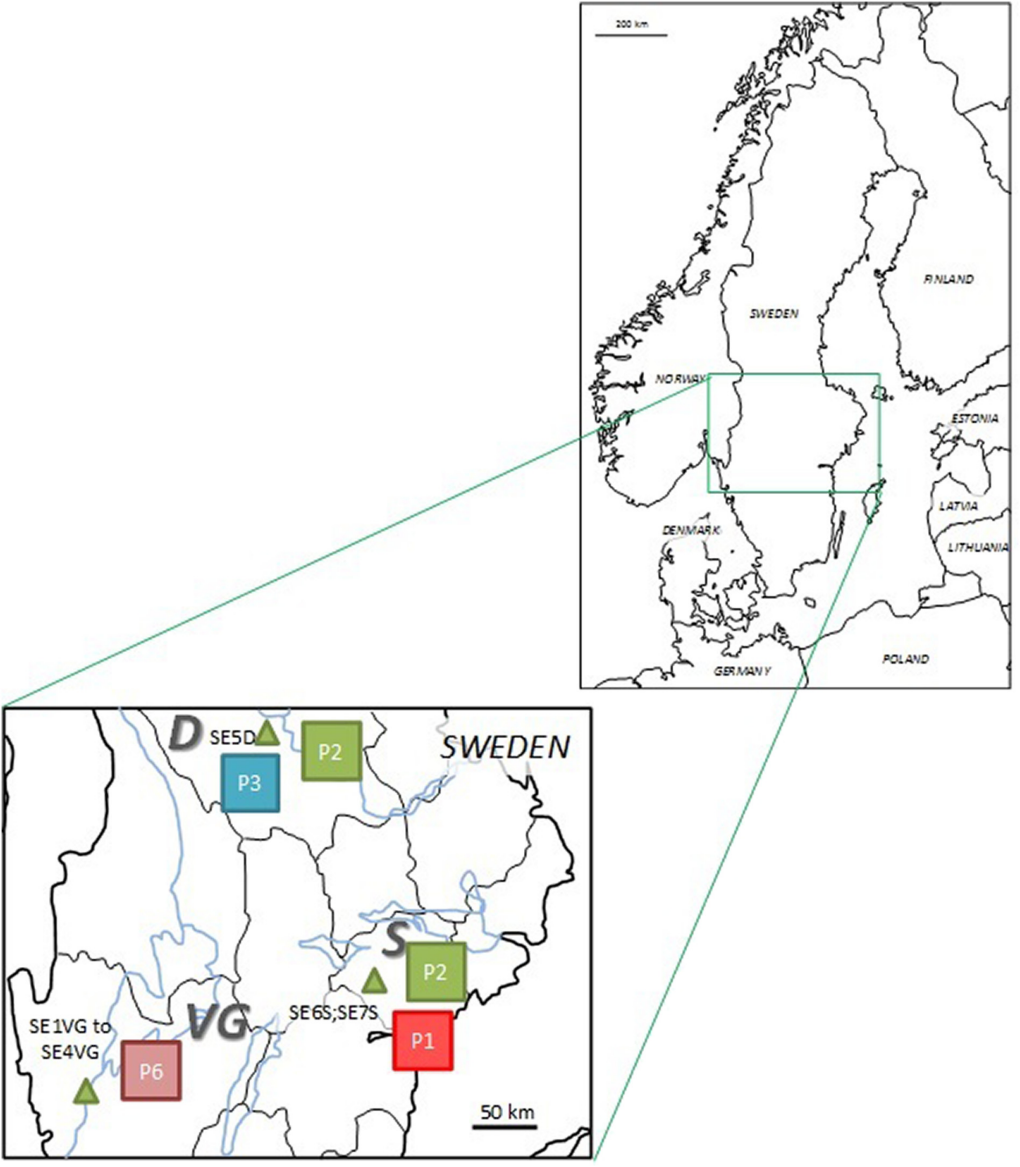
EmsB profiles of *Echinococcus multilocularis* identified in five study areas (marked by triangels) in Denmark (A) and Sweden (B). H: Højer, G: Grindsted, VG: Västra Götaland, D: Dalarna, S:Södermanland. Fox identification numbers are presented next to the EmsB profiles identified from their *E. multilocularis* worms (marked by squares). Note that profiles P1, P2 and P3 are closely related.

In total, six EmsB profiles were identified. One EmsB profile (P1) was common between Denmark and Sweden (six worms from a Danish fox and seven worms from two Swedish foxes). In Denmark, two additional profiles were identified. All worms (*n* = 26) from all foxes (*n* = 7) sampled in the Højer area (H) had identical profiles (P4). In the Grindsted area (G), two different profiles were found: P1 and P5 (Table 1, Fig. 1). In Sweden, a total of four different profiles were found. All worms (*n* = 15) from all foxes (*n* = 4) sampled in the county of Västra Götaland (VG) had the same profile (P6). In the county of Södermanland (S), the profiles P1 and P2 were found in nine worms from two foxes, and one of these foxes harbored worms with both profiles. In the county of Dalarna (D), profile P2 and P3 were detected in one and two worms respectively, recovered from the same fox. The profiles P1, P2 and P3 are closely related, and all of these profiles correspond to G07 from the “ref collection” (Table 1, Figure1) (Knapp et al., 2009).

**Fig. 2:**
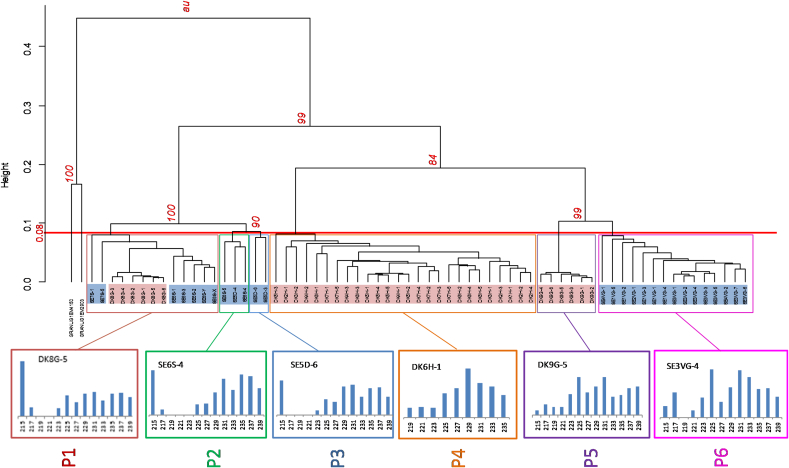
Dendrogram based on EmsB profiles (P1–P6) identified in Swedish (*n* = 27) and Danish (*n* = 38) *Echinococcus multilocularis* worms. The EmsB graphs illustrating the detected profiles are electrophoregrams constructed from raw EmsB data. Each profile represents data from one worm; the bars represent the EmsB alleles with their size in bp (abscissa) and fluorescence height (ordinate). Bootstrap values based on 1000 re-samplings are given next to the nodes, and worms from each fox are indicated according to country and region of origin e.g. DK8G = fox number 8 from Grindsted, Denmark. Sample codes: Country = SE: Sweden; DK: Denmark; Geographical origin: H: Højer; G: Grindsted; VG: Västra Götaland, D: Dalarna, S: Södermanland.

The hierarchical clustering of the worms based on the Euclidean distances is illustrated in Fig. 2. All of the six EmsB profiles identified in this study were in the same size frame (215 to 239 bp) as the European samples from the” ref. collection” (Fig. 2) (Knapp et al., 2017), and all had been previously identified in Europe (Table 2). The corresponding European profiles are detailed in Table 1, Table 2. The “ref collection” data, including the electrophoregram profiles and fragment analysis values are available from the EWET database (Knapp et al., 2017).

**Table 2:** EmsB profiles found in *Echinococcus multilocularis* worms isolated from red foxes in Sweden and Denmark compared to worms from other European countries.

EmsB profile	Swedish worms (no of foxes)	Danish worms (no of foxes)	Corresponding European profile^a^	Reference collection countries^b^, ^c^
P1	7 (2)	6 (1)	G06/G07	F, D, CH, PL, CZ, SL
P2	3 (2)		G05/G07	F, D, CH, PL, CZ, SL
P3	2(1)		G07	F, D, CH, PL
P4		26 (7)	G23/G27/G28	F, D, CH, PL, CZ, SL
P5		6 (1)	G19	F, D, CZ
P6	15 (4)		G20/G21	F, D, CH, CZ, SL

Country codes F: France, D: Germany, CH: Switzerland, PL: Poland, CZ: Czech Republic, SL: Slovakia.

a Knapp et al., 2009.

b Knapp et al., 2007, Knapp et al., 2008, Knapp et al., 2009.

c Umhang et al., 2014.

## 4 Discussion

In the present study, only few EmsB profiles were found in Sweden and Denmark, which is expected in low endemic areas where *E. multilocularis* has presumably been recently established. Similar values of genetic diversity were obtained from peripheral endemic areas in Europe such as Northern and Western France, Northern Poland and Central Slovakia while higher values (>6) were obtained from the historical core areas Switzerland, Jura Swabe (Southern Germany), Northern Austria and Eastern France (Knapp et al., 2009; Umhang et al., 2014). However, the number of different profiles also depends on the sampling intensity, i.e. the number of samples analysed, and if sampling is representative of the area. In this study, 27 worms from seven Swedish foxes and 38 worm from nine Danish foxes were analysed. In comparison, 74 worms from 28 foxes, 46 worms from 10 foxes and 30 worms from seven foxes were analysed from Northern and Western France, Northern Poland and Central Slovakia, respectively (Knapp et al., 2009). Although we conclude that few profiles were found in the present study we cannot claim that the study areas have low genetic diversity based on the present data.

In two areas, one in Denmark (Höjer) and one in Sweden (Västra Götaland), only a single EmsB profile was detected although worms from seven and four foxes, respectively, were analysed. Without prior knowledge of the expected speed of subtype divergence after introduction, the time of introduction of the described profiles cannot be estimated. Furthermore, as dogs travel frequently between Denmark, Sweden and other European countries, subtype diversity does not only depend on subtype divergence. In isolated areas where parasites have limited or no connection with other infected areas the parasite population is expected to be uniform if the area is environmentally homogenous and the host population is not stratified. Yet, this does not explain the low diversity observed in the present areas as they seem to have no obvious natural borders preventing the parasites as well as their hosts from contact with surrounding areas, in contrast to e.g. the autochthonous focus of *E. multilocularis* in Northern Italy (Casulli et al., 2009). It is possible, that the low genetic variation detected in the present study simply reflects the relatively low number of isolates analysed until now. Thus, additional sampling and genetic characterisation of the *E. multilocularis* populations in Scandinavia are clearly warranted.

Slightly more genetic variability was identified in the other three study areas; Södermanland and Dalarna in Sweden and Grindsted in Denmark, where two different profiles were detected in each location. Surprisingly, these findings were done in areas were fewer foxes (maximum of two) were sampled. In Södermanland and Dalarna, the mixed infections were found in dual-infected foxes harbouring worms with different EmsB profiles. This was not the case in Grindsted, Denmark were the different EmsB profiles originated from two mono-infected foxes. Both of these foxes were shot in the same location (Utoft). The fact that different profiles were detected in foxes sharing the same home range suggests that the parasites were introduced separately by animals of diverse origin and/or infected with different *E. multilocularis* profiles. The distance between Utoft, where the foxes were shot and the nearest endemic area in Northern Germany is around 90 km, which is within the known migration distance of red foxes (Meek and Saunders, 2000). However, since relatively few foxes have been analysed so far it is impossible to draw any firm conclusions. Likewise, little effort has been made to isolate *E. multilocularis* in intermediate hosts in Denmark. To our knowledge, the parasite has not yet been detected in rodents in Denmark, and therefore we cannot exclude that the different EmsB profiles may reflect infections contracted in other areas, as foxes are known to migrate over long distances (Meek and Saunders, 2000). Alternatively, the parasites may have been introduced by dogs travelling to high-endemic regions (Osterman Lind et al., 2011; Saeed et al., 2006; Torgerson and Craig, 2009). It should however be highlighted that the three different profiles P1, P2 and P3 identified in our study were very closely related as they all correspond to the same profile (G07) in the” ref. collection”. Furthermore, in Dalarna, despite intensive sampling, only one infected fox has been found and it seems unlikely that this single fox should have been infected with two different subtypes. Furthermore, the only foxes in this study that were infected with more than one subtype (Table 1) were foxes infected with P1 and P2 (SE6S) or P2 and P3 (SE5D). Further studies including additional isolates and more discriminatory methods/several genetic markers may reveal if P1-P3 should in fact be regarded as one genotype. Likewise, some uncertainty exists regarding the possible presence of new EmsB profiles. When using microsatellite analyses, the DNA quality of the sample is crucial because each of the amplified fragments (about 40 copies) are analysed quantitatively. An EmsB profile may be obtained with a DNA sample of low quality, but it will not represent the true structure of the EmsB microsatellite in the sample. Therefore, we took a conservative approach and considered profiles as artefacts (supposedly, mainly due to low DNA quality) if they were very different from previously detected profiles in Europe and only detected in a single worm. Again, additional sampling and typing is necessary to reveal if this was the correct approach.

Only one infected fox was found in Dalarna, whereas several positive samples were detected in the other infected areas in Sweden. Yet, the possibility that the infected fox in Dalarna could have migrated from another infected area is unlikely as young males are the primary driver of migration, whereas adult females (as in this case) are more likely to remain local (Comte et al., 2017). The diversity found in Södermanland, may have been caused by multiple introductions e.g. via hunting dogs infected with the parasite, as hunters from other countries are frequently visiting the area, bringing their own dogs. Although, until 2011, deworming was required for dogs entering Sweden from infected countries, no strict control of compliance has been in place at all ports of entry (Wahlström et al., 2015), and the probability of infected dogs entering Sweden causing repeated introduction and multiple genetic diversity should therefore not be neglected (Wahlström et al., 2012).

In the present study, all the six profiles identified have previously been found in other European countries (Knapp et al., 2009; Knapp et al., 2007), suggesting possible genetic exchange between Sweden/Denmark and other European countries. The low genetic diversity found in our study may indicate that *E. multilocularis* was recently introduced to Sweden and Denmark. Nevertheless, the actual time of introduction cannot be estimated as a molecular clock cannot be deduced from EmsB analysis due to its complex multilocus nature and the general absence of *E. multilocularis* fossil DNA. Based on the epidemiological surveillance data of *E. multilocularis* in both countries with recent first detections and low prevalence levels in foxes the low genetic variation found in our study was expected. Only one of the six profiles identified in the present study was found in both Denmark and Sweden. This suggests that the presence of *E. multilocularis* in these countries cannot be explained solely by transmission of the parasite from one country to the other, even if a potential connection cannot be excluded. If recent introduction has occurred, the most likely sources of infection would be migrating wildlife, for example foxes, or infected domestic animals, for example dogs travelling from, or having visited, high endemic areas. In Denmark, dog travel from other European countries is relatively unrestricted (Fødevarestyrelsen, 2018), and foxes can easily cross the border as there are no natural or artificial boundaries separating the Danish peninsula: Jutland from mainland Europe. In Sweden, foxes may only migrate from the neighboring countries: Finland and Norway; two countries currently considered to be free from *E. multilocularis* (Enemark et al., 2018; Oksanen et al., 2016) with the exception of the Arctic archipelago of Svalbard where presence of *E. multilocularis* has been known since 1999 (Henttonen et al., 2001). However, since 1995, border control of travelling dogs has been less stringent and the risk of introducing *E. multilocularis* via infected dogs is therefore considered to have increased (Wahlström et al., 2012).

## 5 Conclusion

In this study, few genetic profiles were identified in *E. multilocularis* isolated from seven Swedish and nine Danish foxes, which may indicate recent introduction of the parasite to these countries. The description of the genetic diversity of *E. multilocularis* via EmsB profiles performed in the present study is expected to be useful for comparisons with future samples in order to increase our understanding of the epidemiologic situation in Scandinavia and the general expansion of *E. multilocularis* in Europe.
